# 784. Efficiency of Quaternary Ammonium Disinfectants against SARS-CoV-2 and Coronavirus 229E

**DOI:** 10.1093/ofid/ofab466.981

**Published:** 2021-12-04

**Authors:** Charles P Gerba

**Affiliations:** University of Arizona, Tucson, AZ

## Abstract

**Background:**

The recent pandemic of CoVid19 has increased our need to assess the impact of disinfectants on the inactivation of human coronaviruses. The goals of this study were 1) quantify the disinfection of SARS-CoV-2 and human coronavirus 229 inactivations by various quaternary ammonium formulations, and 2) demonstrate the impact of disinfectants on preventing fomite-to-finger transfer of coronaviruses.

**Methods:**

We compared the inactivation of both SARS-Covid -2 and coronavirus 229E suspended in 5% fetal calf sera and dried on both metal and plastic surfaces. In addition, studies were conducted with a silinated quaternary ammonium compound that left a residual on the surface. Studies were also conducted on the finger transfer of coronavirus from various surfaces. The virus was allowed to dry on the surface for 30 minutes, then a transfer was conducted by placing the finger pad directly onto the contaminated surface. The finger was tested for the virus. The study was then repeated with virus-contaminated porcelain surfaces that were sprayed with a quaternary product or placed on a surface with a quaternary ammonium compound that left a residual.

**Results:**

Several readily available quaternary ammonium formulations were evaluated and proved to be effective with greater than a 99.9% reduction in titer after drying on both metal and plastic surfaces. In addition, a silinated quaternary ammonium compound that left a residual on the surface was capable of inactivating SARS-CoV-2 for at least seven days after application. Studies on the finger transfer of coronavirus from various surfaces showed that the amount of virus transfer to the finger varied from 0.46 to 49.0% depending upon the surface. Little or no virus transfer occurred from treated surfaces compared to the untreated controls. In addition, coronavirus 229E appears to be a good model for use in disinfection assessments for SARS-CoV-2.

**Conclusion:**

Our results demonstrate that various quaternary ammonium disinfectant formulations are effective against human coronaviruses. Finger transfer tests showed that transmission of coronavirus from surfaces can be prevented, reducing the risk of fomite transmission. Coronavirus 229E appears to be a good model for use in disinfection assessments for SARS-CoV-2.

**Disclosures:**

**Charles P. Gerba, Ph.D.**, **Allied Biosciences** (Grant/Research Support)**Behr** (Grant/Research Support)**Corning Inc.** (Grant/Research Support)**PPG** (Grant/Research Support)**Procter and Gamble** (Other Financial or Material Support, donation)**Rickett and Coleman** (Grant/Research Support)

